# The conserved p.Arg108 residue in S1PR2 (DFNB68) is fundamental for proper hearing: evidence from a consanguineous Iranian family

**DOI:** 10.1186/s12881-018-0598-5

**Published:** 2018-05-18

**Authors:** Michaela A. H. Hofrichter, Majid Mojarad, Julia Doll, Clemens Grimm, Atiye Eslahi, Neda Sadat Hosseini, Mohsen Rajati, Tobias Müller, Marcus Dittrich, Reza Maroofian, Thomas Haaf, Barbara Vona

**Affiliations:** 1 Institute of Human Genetics, Julius Maximilians University, Würzburg, Germany; 2 Department of Medical Genetics, School of Medicine, Mashhad University of Medical Sciences, Mashhad, Iran; 3 Medical Genetics Research Center, Faculty of Medicine, Mashhad University of Medical Sciences, Mashhad, Iran; 4 Department of Biochemistry, Biocenter, Julius Maximilians University, Würzburg, Germany; 5 Department of Otorhinolaryngology-Head and Neck Surgery, Faculty of Medicine, Ghaem Educational Hospital, Mashhad University of Medical Sciences, Mashhad, Iran; 6 Institute of Bioinformatics, Julius Maximilians University, Würzburg, Germany; 7 Genetics and Molecular Cell Sciences Research Centre, St George’s, University of London, Cranmer Terrace, London, SW17 0RE UK

**Keywords:** 3D modeling, Autosomal recessive non-syndromic hearing loss, DFNB68, Mixed hearing loss, *S1PR2*, Whole exome sequencing

## Abstract

**Background:**

Genetic heterogeneity and consanguineous marriages make recessive inherited hearing loss in Iran the second most common genetic disorder. Only two reported pathogenic variants (c.323G>C, p.Arg108Pro and c.419A>G, p.Tyr140Cys) in the *S1PR2* gene have previously been linked to autosomal recessive hearing loss (DFNB68) in two Pakistani families. We describe a segregating novel homozygous c.323G>A, p.Arg108Gln pathogenic variant in *S1PR2* that was identified in four affected individuals from a consanguineous five generation Iranian family.

**Methods:**

Whole exome sequencing and bioinformatics analysis of 116 hearing loss-associated genes was performed in an affected individual from a five generation Iranian family. Segregation analysis and 3D protein modeling of the p.Arg108 exchange was performed.

**Results:**

The two Pakistani families previously identified with *S1PR2* pathogenic variants presented profound hearing loss that is also observed in the affected Iranian individuals described in the current study. Interestingly, we confirmed mixed hearing loss in one affected individual. 3D protein modeling suggests that the p.Arg108 position plays a key role in ligand receptor interaction, which is disturbed by the p.Arg108Gln change.

**Conclusion:**

In summary, we report the third overall mutation in *S1PR2* and the first report outside the Pakistani population. Furthermore, we describe a novel variant that causes an amino acid exchange (p.Arg108Gln) in the same amino acid residue as one of the previously reported Pakistani families (p.Arg108Pro). This finding emphasizes the importance of the p.Arg108 amino acid in normal hearing and confirms and consolidates the role of *S1PR2* in autosomal recessive hearing loss.

**Electronic supplementary material:**

The online version of this article (10.1186/s12881-018-0598-5) contains supplementary material, which is available to authorized users.

## Background

Hereditary hearing loss (HL) is present in one to two per 1000 newborns [1] in developed countries and follows an autosomal recessive pattern of inheritance in approximately 70% of cases [2]. A genetic etiology is estimated to occur in 50%-60% of all cases [1, 2]. In Iran, 1 out of 166 individuals suffers from HL [3], a prevalence that is largely attributed to a significant proportion of consanguineous marriages [4]. Around 80% of autosomal recessive HL genes have been discovered by investigating consanguineous families [5, 6].

*S1PR2* (OMIM: 605111) encodes a 353 amino acid sphingosine-1-phosphate receptor 2 (S1PR2) and maps to the DFNB68 (OMIM: 610419) locus on chromosome 19p13.2 [7]. Variants in *S1PR2* have been implicated in congenital profound sensorineural HL without vestibular defects in two Pakistani families with recessive HL [8]. Linkage mapping and whole exome sequencing in these families identified homozygous c.323G>C (p.Arg108Pro) and c.419A>G (p.Tyr140Cys) mutations in *S1PR2*.

*S1PR2* is part of the sphingosine-1-phosphate signaling (S1P) pathway and is required for normal auditory function [9]. Detailed expression analysis localized S1pr2 to the cell bodies of inner and outer hair cells, the stria vascularis, spiral ligament fibrocytes, as well as spiral ganglion cells in the mouse [9]. S1pr2 defects elicit abnormal endocochlear potential (EP) measurements, morphological changes in the stria vascularis and secondary hair cell degeneration attributed to abnormal EP [9]. To date, one spontaneous missense and three *S1pr2* knockout mouse models have been described [9–12]. All three knockout mouse models showed profound-to-complete deafness at one month of age and progressive vestibular defects [10–12]. Missense mutants presented rapidly progressive HL with reduced EP followed by a loss of cochlear hair cells [9]. No other malformations were identified in these mouse models, thus revealing a role of *S1pr2* in hearing function without other organ system involvement. Additionally, zebrafish morphants demonstrated abnormal otic vesicle and lateral line morphology, supporting a key role of this gene in auditory maintenance [13].

In our study, whole exome sequencing of 116 hearing-associated genes disclosed the third novel pathogenic variant in *S1PR2* in a proband from a large consanguineous Iranian family (E30) segregating bilateral, severe-to-profound recessive HL. We describe a homozygous pathogenic variant in *S1PR2* that was identified in the first family outside Pakistan. Interestingly, this novel pathogenic variant affects the same amino acid residue as one of the previously reported mutations [8]. Clinical evaluation disclosed the first reported mixed HL in an affected individual, which suggests an expanded clinical outcome of individuals with mutations in *S1PR2*. Additionally, we model the identified amino acid residue exchange at the p.Arg108 position of S1PR2 and highlight the consequences of spatial disruption of this key amino acid with neighboring amino acid residues.

## Methods

### Clinical examination and family recruitment

**Fig. 1:**
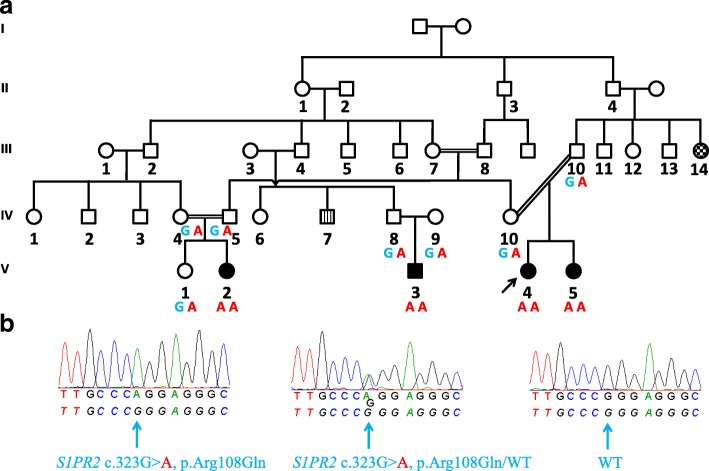
Pedigree and segregation of the *S1PR2* c.323G>A variant in family E30. **a** A five generation family with four affected individuals with HL and seven unaffected family members was subjected to segregation testing. The wild type allele is marked in blue, the variant in red. The segregation results are shown below each individual who was tested. Parental consanguinity is noted for V-2, as well as V-4 and V-5. Affected individuals with HL are colored in black and unaffected individuals are colorless. An individual with epilepsy is marked with a checkered pattern (III-14) and an individual with polydactyly is indicated with a striped pattern (IV-7). **b** Sanger sequence chromatograms of the *S1PR2* c.323G>A pathogenic variant in homozygous (left) and heterozygous (center) orientations. An illustrative WT Sanger sequence chromatogram is also included (right). The reference sequence is shown at the bottom, whereas the individual nucleotide sequence is shown at the top. A blue arrow indicates the c.323 position

The four affected individuals (V-2, V-3, V-4, V-5) of family E30 (Fig. 1a) underwent multiple audiological assessments that adhered to recommendations described in Mazzoli et al., 2003 [14]. The younger individuals (<10 years of age) (V-4, V-5) were subjected to auditory brainstem response (ABR) and auditory steady state response (ASSR) testing, as well as transient-evoked otoacoustic emission (TEOAE) measurements. The HL of the oldest affected individual (V-2) was investigated by pure-tone audiometry.

### Whole exome sequencing

Genomic DNA (gDNA) was extracted from peripheral blood leukocytes from the proband (V-4, Fig. 1a) and her family members that included seven normal hearing and three hearing impaired individuals. Mutations and deletions were excluded in *GJB2* and *STRC*, respectively. Proband gDNA was subjected to whole exome sequencing. The exome library of the proband was prepared using the Nextera Rapid Capture Enrichment kit according to manufacturer’s instructions (Illumina, San Diego, CA, USA). Subsequently, the samples were 2x76 bp paired end sequenced on a NextSeq500 benchtop sequencer (Illumina, San Diego, CA, USA) with a v2 reagent kit and mapped to the human genome reference GRCh37 (hg19).

### *In silico* analysis of whole exome data

Data were analyzed using GensearchNGS software (PhenoSystems SA, Wallonia, Belgium) and an in-house bioinformatics pipeline. Both bioinformatics platforms applied variant filtering against minor allele frequency >0.01 and alternate allele present at >20%. Analysis focused on missense, frameshift, nonsense and splice site variants. Pipeline data were analyzed based on the Genome Analysis Toolkit (GATK) [15]. This includes Burrows-Wheeler Alignment-based read alignment to the human genome [16] according to GATK best practice recommendations [17]. Quality filtering was performed based on the VQSLOD score. An in-house allele count filter removed variants that were likely technical artifacts, which included an allele count of up to 10%. The filtering was performed in a population-specific manner, which included data from the Greater Middle East Variome Project [18]. After filtering against population frequency, variants flagged as low quality were manually re-checked to avoid missing a potentially causative variant in a low quality set. Variant prioritization followed the use of multiple prediction algorithms such as CADD [19], PolyPhen-2 [20], SIFT [21], and MutationTaster [22], as well as variant adjustment according to the Deafness Variation Database (DVD) (containing >7000 pathogenic or likely pathogenic variants) [23] and ClinVar [24]. Whole exome copy number variation calling was performed using the eXome Hidden Markov Model (XHMM, version 1.0) approach as detailed by Menachem Fromer and Shaun M. Purcell [25]. An *in silico* gene panel including 116 HL-associated genes [see Additional file 1] was used to guide the exome data analysis. Parental consanguinity of the proband (V-4) suggested an autosomal recessive pattern of inheritance (Fig. 1a) and filtering included analysis of homozygous and compound heterozygous variants. These variants were further classified using Alamut Visual version 2.7.1 (Interactive Biosoftware, Rouen, France), as well as databases such as OMIM, ExAC, gnomAD [26] and HGMD [27]. Additionally, variants were also investigated by splice predictors (SpliceSiteFinder-like, MaxEntScan, NNSPLICE, Genesplicer and Human Splicing Finder), which are included in Alamut Visual biosoftware.

### Sanger sequencing validation and segregation analysis

Validation and segregation testing of the *S1PR2* (NM_004230.3) variant in the single exon gene was performed using PCR-amplified gDNA from the proband (V-4) and her family members (V-1, V-2, V-3, V-5, IV-4, IV-5, IV-8, IV-9, IV-10 and III-10, Fig. 1a) using standard cycling conditions with a forward (5’-AATTGAATCTCAGCCCATCC-3’) and reverse (5’-TAATGCTTGGCGTAGAGAGG-3’) (Metabion, Martinsried, Germany) primer. Primers were designed using Primer3 [28]. The 700 bp amplicons were Sanger sequenced with an ABI 3130xl 16-capillary sequencer (Life Technologies, Carlsbad, CA, USA) and the data were aligned against human reference genome NCBI build GRCh37p10 and analyzed using Gensearch 4.3 software (PhenoSystems SA, Wallonia, Belgium).

### 3D modeling

The amino acid sequences of the wild type (WT) S1PR2 protein (NP_004221.3) shared 55% sequence identity with the S1PR1 protein (NP_001307659.1). A homology model of the S1PR2 p.Arg108Gln exchange based on the available crystal structure of S1PR1 (PDB ID: 3v2y) [29] was created using the program Phyre2 (Homology/analogY Recognition Engine version 2.0) [30] and analyzed by the programs Chimera [31] and PyMol^TM^ (DeLano Scientific LLC). The S1PR1 model with the ligand ML056, an analog to the natural S1P (PDB: 3v2y) [29] was used to investigate the impact of the arginine to glutamine substitution on the ligand-receptor interaction. Herein, the substitution was generated manually by *Coot* [32] and analyzed in combination with the WT structure of S1PR1 by PyMol^TM^.

## Results

### Family history

A five generation family E30 of Iranian descent presented four individuals (V-2, V-3, V-4 and V-5) with autosomal recessive HL. Fig. 1a shows an abbreviated pedigree that includes all individuals with HL. Additional phenotypes such as polydactyly (3 individuals), epilepsy (4 individuals), and intellectual disability (2 individuals) are also noted in other branches of this family, with two individuals shown in Fig. 1a, but these additional phenotypes were not observed in the individuals with HL. All affected children in the fifth generation underwent clinical examination.

**Fig. 2:**
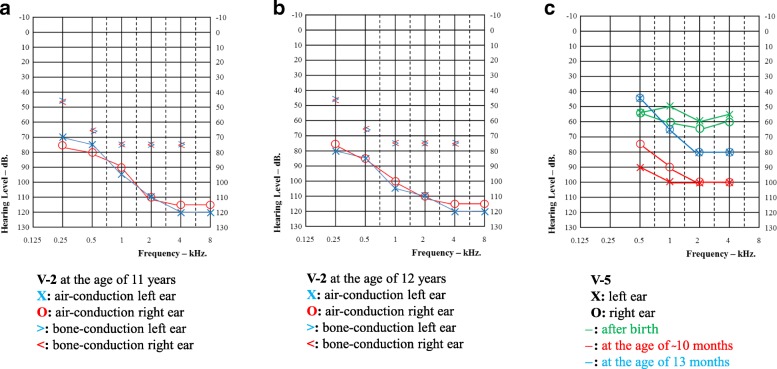
Auditory evaluations of V-2 and V-5. Circles and crosses denote thresholds for the left and right ear, respectively. **a** Pure-tone audiograms of V-2 at the age of 11 and **b** 12 years. Air-conduction thresholds for right and left ears are represented with circles and crosses, respectively. Bone-conduction is represented > and < for left and right ears, respectively. **c** ASSR measurements for V-5. ASSRs were measured at three different time points: first after birth (green), at the age of 10 months (red) and at the age of 13 months (blue)

The affected individuals presented HL with a prelingual onset ranging from birth to the first year of life. The HL is bilateral and severe-to-profound (Fig. 2a-b). Individual V-2 is currently 15 years old. This individual underwent pure-tone air- and bone-conduction audiometry at 11 and 12 years of age and showed sloping audiograms with stable HL (Fig. 2a-b). Interestingly, mixed HL is evident by an air-bone gap in both pure-tone audiograms on record, suggesting both middle and inner ear defects. Tympanometry of V-2 revealed a normal A type curve (data not shown). Radiological imaging (CT and MRI) has not been performed. Almost all affected individuals present profound sensorineural HL except the sister of the index (V-5) with severe HL (Table 1).

**Table 1:** Characterization of HL in each affected individual in family E30

Hearing loss	V-2	V-3	V-4	V-5
Approximate age of onset	Before 1 year old	Before 1 year old	Before 1 year old	Congenital
Age at auditory examination	1.6 years	~ 2 years	7 months	10 days
Current age	15 years	4.4 years	8 years	2 years
Type	Mixed	Sensorineural	Sensorineural	Sensorineural
Laterality	Bilateral/symmetric	Bilateral/symmetric	Bilateral/symmetric	Bilateral/symmetric
Suspected intrafamilial variability	No	No	No	Yes
Degree	Profound	Profound	Profound	Severe
Non-syndromic	Yes	Yes	Yes	Yes

ABRs were abnormal for individuals V-4 and V-5. ASSR thresholds for the index patient (V-4) were completely absent across all frequencies in the left ear and were detected at 90 dB each at 0.5 and 1 kHz and 110 dB each at 1 and 2 kHz. TEOAEs in the proband (V-4) were absent with 3% and 6% reproducibility for right and left ears, respectively. However, the ASSR thresholds for her younger sister (V-5) showed progressive decline (Fig. 2c) between 10 and 13 months of age with absent TEOAEs. No tinnitus or vestibular symptoms were reported in the affected individuals.

### Identification and characterization of the c.323G>A variant

The proband V-4 was subjected to whole exome sequencing and bioinformatics analysis that included 116 HL-associated genes. Bioinformatics analysis revealed over 1600 unfiltered variants that were filtered after pipeline analysis to three heterozygous variants and one homozygous variant in autosomal recessive HL genes, as well as one heterozygous variant in an X-linked recessive HL gene and two heterozygous variants in autosomal dominant HL genes. A homozygous c.323G>A, p.Arg108Gln variant in the gene *S1PR2* (DFNB68) was identified using both bioinformatics platforms. All other variants were prioritized as benign or inadequate to cause HL in this family. Copy number variation analysis in the HL-associated genes was negative. Upon closer inspection of the exome dataset using GensearchNGS, the *S1PR2* c.323G>A pathogenic variant was found to reside in a 1.65 Mb run of homozygosity on chromosome 19p13.2 (coordinates chr19:9,025,652-10,676,487). An in-house exome database filtered for *S1PR2* variants in the nearly 300 families with HL who are included in our on-going exome projects did not disclose additional putative pathogenic variants in *S1PR2*.

*S1PR2* had a mean depth of 44-fold and was covered with over 80 reads at the c.323 position. Overall, 95% of all HL gene capture regions were covered with a mean depth of at least 10×. 83.4% of *S1PR2* achieved a 10-fold coverage. The c.323G>A, p.Arg108Gln variant results in a likely pathogenic amino acid exchange according to pathogenicity prediction tools (CADD, MutationTaster, SIFT, PolyPhen-2). The variant was not archived in ClinVar, DVD, HGMD, ExAC and gnomAD databases (Table 2).

**Table 2:** Comparison of all known human *S1PR2* pathogenic variants

	Family		
Prediction of Variation	E30	DEM4154	PKDF1400
hg19 position, Chr.19	10,335,259	10,335,259	10,335,163
cDNA change	c.323G>A	c.323G>C	c.419A>G
Amino acid change	p.Arg108Gln	p.Arg108Pro	p.Tyr140Cys
ExAC	0	0	0
gnomAD	0	0	0
CADD	34	21.7	22.9
MutationTaster	Disease causing	Disease causing	Disease causing
SIFT	Deleterious	Deleterious	Tolerated
PolyPhen-2	Probably damaging	Probably damaging	Probably damaging
ClinVar	No entry	Pathogenic	Pathogenic
DVD	No entry	No entry	No entry
HGMD	No entry	Hearing impairment, autosomal recessive	Hearing impairment, autosomal recessive

This exchange affects a highly conserved nucleotide (phyloP: 5.77, reference -14.1 to 6.4) and amino acid up to tetraodon (considering 11 species). The physiochemical difference between the arginine and glutamine amino acid residue exchanges is small (Grantham distance: 43, range 0 to 215) (Alamut Visual version 2.7.1). Nevertheless, the c.323G>A provokes a small change in the 3’ splice performance in two of the five splice prediction tools in Alamut (data not shown). A novel splice site that is the result of the c.323G>A variant creates a novel 3’ splice acceptor site with a splicing prediction score of 87.1 (reference range: 0-100) in Human Splicing Finder and 2 (reference range: 0-16) in MaxEntScan. The functional impact of this predicted cryptic splice site activation was not tested. Furthermore, the variant affects one of the 67 CpG sites in the single coding exon of *S1PR2*. The variant was validated by Sanger sequencing (Fig. 1b) and segregates appropriately supporting a likely pathogenic outcome (Fig. 1a). All affected children have the homozygous pathogenic variant, whereas their parents and an unaffected sibling of V-2 are heterozygous.

Interestingly, the same c.323 position has been reported with a different homozygous nucleotide exchange (c.323G>C) in a family with congenital profound sensorineural HL and lower limb deformities [8]. This variant results in a p.Arg108Pro amino acid exchange and exhibits a moderate physiochemical difference (Grantham distance: 103), but is not predicted as impacting splicing. The CpG site shifts one position in *S1PR2*.

### Homology modeling of the S1PR2 p.Arg108 pathogenic variant

**Fig. 3:**
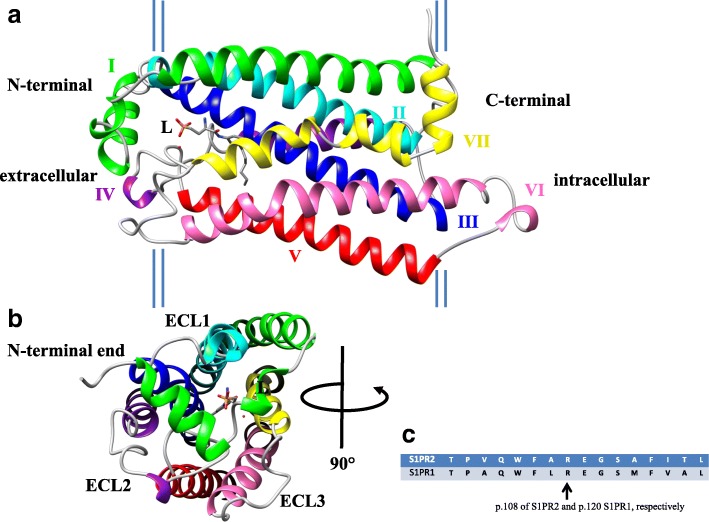
3D homology model of WT S1PR2 protein structure. **a** Description of the single helices. Helix I includes the N-terminal end and is colored in green. Helix II (turquoise) and helix III (blue) form ECL1, helix IV (purple) and helix V (red) form ECL2, and helix VI (pink) and helix VII (yellow) form ECL3. L represents the ligand ML056, which mirrors the S1P ligand. **b** View of S1PR2 from the extracellular side. The ECLs and N-terminal end are marked. L describes the ligand ML056. **c** Partial human protein sequence alignments of S1PR2 and S1PR1 including p.108 and p.120, respectively

Based on the S1PR1 crystal structure, we designed a homology model of S1PR2 to compare the structural outcome of p.Arg108Gln with the previously published p.Arg108Pro amino acid exchange. The WT protein structure has seven transmembrane helices (TM1-7). Together with extracellular loops 1 and 2 (ECL1 and ECL2), the N-terminal helix I (Fig. 3a, green) creates the ECL-binding pocket receptor for S1P or the analog ML056 shown as L in Fig. 3a-b [29]. Each ECL bridges two transmembrane helices. There are three ECLs: ECL1 bridges helices II and III, ECL2 connects helices IV and V, and ECL3 links helices VI and VII (Fig. 3b). Transmembrane helix III (TM3) (Fig. 3a, blue) harbors residues Arg108 and Glu109, which are both identical to the S1P-binding amino acid residues Arg120 and Glu121 of the S1PR1 receptor (Fig. 3c) [8, 29]. As previously described, Arg108 directly forms a salt bridge to the negatively charged phosphate head of S1P [8, 29, 33].

**Fig. 4:**
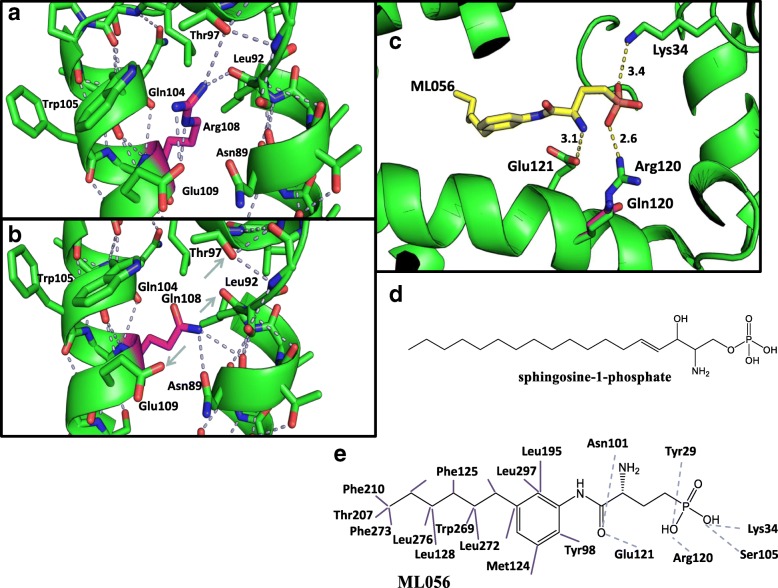
3D and H-bond analysis of S1PR2 amino acid residue exchanges at position 108. H-bonds are predicted by the modeling program PyMol. The amino acid in position 108 is marked in magenta. H-bonds are marked in grey dashes. The interacting amino acids are labeled. **a** WT arginine and **b** mutant glutamine at position 108. Small grey arrows present missing H-bonds. **c** Crystal structure of S1PR1 (3v2y). The p.Arg120Gln substitution (magenta) is described in relation to the ligand ML056. The amino acids p.121Glu and p.120Arg build ionic and H-bonds (yellow dashes) with the ligand ML056 (yellow structure). The Å-distances are listed at the yellow dashes. **d** Chemical structure of the sphingosine-1-phosphate. **e** The chemical structure of the S1P analog, ML056. The dashed grey lines show the polar interactions between amino acids of S1PR1 and the ligand and the purple lines represent the hydrophobic interactions of ML056 with the amino acids of S1PR1 [29]

In a resting state, without interaction of S1P, Arg108 coordinates a hydrogen bond (H-bond) network including residues Leu92, Thr97, Gln104, Trp105 and Glu109 (Fig. 4a). The substitution of arginine amino groups by a glutamine moiety likely does not strongly impact H-bonding [8]. While its interactions with Gln104 and Trp105 carboxyl groups are maintained, the H-bonds to Thr97, Leu92 and Glu109 are lost (Fig. 4b, grey arrows). Instead, the glutamine amino group binds to the carboxyl group of Asn89 (Fig. 4b). In the active state (bound S1P ligand, Fig. 4d), mirrored by a S1PR1 model with bound ML056 ligand (Fig. 4e), a S1P analog, the substitution p.120Arg>Gln destroys the necessary interaction of p.120 with the phosphate head of ML056 (Fig. 4c). The glutamine (magenta, Fig. 4c) is unable to form an ionic bond with the ligand ML056, which prevents proper ligand binding and leads to disturbed interaction with ML056. In conclusion, based on the similarity of S1PR2 and S1PR1, as well as the high conservation of the amino acids p.120Arg and p.121Glu, which are p.Arg108 and p.Glu109 in S1PR2 (Fig. 3c), respectively [8], we suggest that the p.Arg108 position plays a key role in the ligand receptor interaction, which is disturbed by the exchange of Arg to Gln at position p.108.

## Discussion

### Prevalence of inherited HL in Iran

The prevalence of inherited HL in Iran is higher than in Western countries [34], which is attributed to high rates of parental consanguinity and the large fraction of HL that follows an autosomal recessive mode of inheritance [4, 35]. An estimated 1 out of 332 Iranians have inherited HL assuming that half of HL in Iran is due to a genetic etiology [3]. Diagnostic rates in Iranian HL patients are presently around 70% [36, 37]. Interestingly, the Iranian population is quite heterogeneous [36], which explains why the novel c.323G>A *S1PR2* variant is involved in this first reported observation of *S1PR2* HL in a large Iranian family. Nevertheless, variants in *S1PR2* seem to be quite rare considering that no further putative pathogenic alleles were present in our cohort of approximately 300 families including over 90 Iranian families.

### Function of the S1PR2 receptor and the c.323G>A substitution

S1P, a lysophospholipid intermediate, binds to the N-terminal ECL surface of S1PR2 [9] and phosphorylates the MAPK/ERK pathway affecting transcription or translation of inner ear proteins, as well as phosphorylation of ERM (ezrin, radixin and moesin) proteins for their activation in cytoskeletal morphology [8, 38, 39]. This interaction plays a major role in the stria vascularis, where S1PR2 is fundamental for EP generation that relies on coordinated separation, diffusion, and transport of K^+^ for electrochemical gradient maintenance [40, 41]. Pathogenic variants in *S1PR2* are thought to affect S1P ligand activation or non-formation of the S1PR1-G protein complex [8].

The identified c.323G>A (p.Arg108Gln) pathogenic variant in family E30 most likely affects the function of S1PR2. In a resting state, the novel amino acid residue exchange could potentially influence the receptor by loss or gain of specific H-bonds (Fig. 4a-b). Whereas the previously described proline exchange degrades the H-bond network and instead forms a single H-bond with Ser111 [8], the replacement of arginine with glutamine suggests a similar effect but with a new H-bond to Asn89. However, in an active state, the exchange of p.Arg108 to p.Gln108 leads to destruction of the S1P-S1PR2 interaction, well-characterized by the S1PR1-ML056 model (Fig. 4c-e). A possible rescue of residual S1PR1 function is proposed by the ionic bond to p.Lys34 (Fig. 4c). Based on the high homology of both receptors, we would expect the same effect in S1PR2 (Fig. 3c). In conclusion, the absence of the most important binding to p.Arg108 may affect the S1PR2 binding efficiency of S1P, thereby controlling protein signaling efficiency [29] and highlights p.Arg108 as a key player in the S1PR2-S1P interaction.

Pathological changes in the stria vascularis by mutated *S1pr2* can be seen at postnatal day 14 of knockout mice. By postnatal day 22, hair cell and spiral ganglion neuron degeneration can be observed and corresponds with profound deafness [8]. Progressive deterioration or absence of utricular and saccular otoconia were noted with advancing age in knockout mice [8], whereas a missense mutant line showed normal vestibular function and overlapping phenotypic similarities [9]. A knockdown of the zebrafish *s1pr2* homolog revealed abnormalities in the otic vesicle, as well as the lateral line hair cells and neuromasts. Morphant semicircular canals showed structural defects. The hair cell and otic vesicle pathologies mirrored the cochlear and vestibular deficits described in the various mouse models [8, 13].

Interestingly, *S1pr2* is expressed in the inner and outer hair cells of mice but hair cell morphology is normal at hearing onset in the many mouse mutants published to date [9–12]. Hair cell decline in *S1pr2* mutant mice may be the result of two pathological processes that are due to disruption of EP and an accumulation of reactive oxygen species that lead to a degeneration of the cochlea. The progressive hair cell dysfunction is likely a secondary effect; however, whether mutated S1PR2 directly causes pathological lesions in hair cells cannot be excluded [9, 42]. Whether a single CpG loss negatively impacts gene expression is unclear. Expression would play a role in the signaling of S1PR2-S1P interaction [8, 38].

### Clinical manifestations of the c.323G>A, p.Arg108Gln variant

The severe-to-profound HL in the affected individuals of family E30 was apparent at an early age. This severe HL is similar to families DEM4154 and PKDF1400 (Table 1) [8]. Vestibular function is intact similar to the missense mouse line. This suggests a possible residual function of S1P-S1PR2 signaling by a different ligand-receptor interaction and that the otoconia development and function in the vestibular complex may be preserved [11, 43]. Nevertheless, the HL in family DEM4154 [8] is more severe than in family E30 which is likely due to the different substitution. Unlike family DEM4154, the family we report here has no limb malformations supporting the role of S1PR2 in non-syndromic HL and the hypothesis of Santos-Cortez et al. that the limb malformations seen in family DEM4154 is due to a different underlying genetic cause. In the missense mouse model, there were no changes in the middle ear, ossicles, or inner ear [9]. Individual V-2 has mixed HL and is also phenotypically unique compared to her cousins. We cannot exclude that her cousins may also present with mixed HL when they are the same age. Similarly, we cannot exclude a possible mixed HL in other affected individuals at this time due to missing bone-conduction thresholds. Secondary genetic factors and modifying alleles cannot be excluded as influencing different phenotypic outcomes in this family. Another possibility contributing to mixed HL could be related to S1PR2 signaling because S1PR2 and S1P also play a role in bone osteogenesis. If a middle ear malformation is present, this mixed HL could be caused by abnormal S1P/S1PR2 signaling in the ROCK signaling pathway, which triggers bone formation [44]. Nevertheless, it cannot be excluded that the conductive component could be a potential incidental finding.

ABR measurements are effective for early detection of EP triggered HL. Several relevant genes such as *S1PR2*, *SLC26A4*, and *KCNJ10* underlie HL due to EP abnormalities [9, 45, 46]. Similar to S1PR2, SLC26A4 maintains the EP by secreting HCO_3_^-^ ions into endolymph [45] and KCNJ10 is a K^+^ transporter which is necessary for EP [46]. Further research into S1PR2 is needed to direct therapeutic target development [8, 42]. Early therapy to restore functional S1PR2 may reduce cochlear degeneration and preserve hearing.

## Conclusions

DFNB68-related HL has been previously reported in two Pakistani families with prelingual, severe-to-profound sensorineural HL. We describe the third DFNB68 family worldwide and the first consanguineous Iranian family identified that emphasizes the rarity of HL due to pathogenic variants in *S1PR2*. The present study reported the first occurrence of mixed HL. Interestingly, the recurrently affected p.Arg108 amino acid residue was involved in both Pakistani and Iranian families which underscore the importance of this amino acid in the function of S1PR2. In conclusion, this study sheds light on the potential mechanism that causes HL due to amino acid residue exchanges of S1PR2 and further confirms *S1PR2* as a gene critical for normal hearing function.

## Additional file

Additional file 1: List of exome panel evaluated genes with OMIM number. (DOCX 21 kb)

## Abbreviations

ABR: Auditory brainstem response
ASSR: Auditory steady state response
ECL: Extracellular loop
EP: Endocochlear potential
ERM: Ezrin, radixin and moesin
GATK: Genome Analysis Toolkit
gDNA: Genomic DNA
H-bond: Hydrogen bond
HL: Hearing loss
S1P: Sphingosine-1-phosphate
TEOAE: Transient-evoked otoacoustic emission
WT: Wild type
