# Supplementary material for: The conserved p.Arg108 residue in S1PR2 (DFNB68) is fundamental for proper hearing: evidence from a consanguineous Iranian family
Source: BMC Med Genet. 2018 May 18;19:81. doi: 10.1186/s12881-018-0598-5 (PMC5960148; doi:10.1186/s12881-018-0598-5)
Supplement: Supplementary file 1 — List of exome panel evaluated genes with OMIM number. (DOCX 21 kb) [file 12881_2018_598_MOESM1_ESM.docx]

| **Gene** | **OMIM number** |
| --- | --- |
| *MCM2* | 116945 |
| *MET* | 164860 |
| *MIR96* | 611606 |
| *MITF* | 156845 |
| *MSRB3* | 613719 |
| *MYH14* | 608568 |
| *MYH9* | 160775 |
| *MYO15A* | 602666 |
| *MYO3A* | 606808 |
| *MYO6* | 600970 |
| *MYO7A* | 276903 |
| *NARS2* | 612803 |
| *OSBPL2* | 606731 |
| *OTOA* | 607038 |
| *OTOF* | 603681 |
| *OTOG* | 604487 |
| *OTOGL* | 614925 |
| *P2RX2* | 600844 |
| *PAX3* | 606597 |
| *PCDH15* | 605514 |
| *PDZD7* | 612971 |
| *PNPT1* | 610316 |
| *POU3F4* | 300039 |
| *POU4F3* | 602460 |
| *PRPS1* | 311850 |
| *PTPRQ* | 603317 |
| *RDX* | 179410 |
| *ROR1* | [602336](https://www.ncbi.nlm.nih.gov/omim/602336) |
| *S1PR2* | 605111 |
| *SERPINB6* | 173321 |
| *SIX1* | 601205 |
| *SLC17A8* | 607557 |
| *SLC22A4* | 604190 |
| *SLC26A4* | 605646 |
| *SLC26A5* | 604943 |
| *SLC44A4* | 606107 |
| *SMPX* | 300226 |
| *SNAI2* | 602150 |
| *SOX10* | 602229 |
| *STRC* | 606440 |
| *SYNE4* | 615535 |
| *TBC1D24* | 613577 |
| *TECTA* | 602574 |
| *TJP2* | 607709 |
| *TMC1* | 606706 |
| *TMEM132E* | 616178 |
| *TMIE* | 607237 |
| *TMPRSS3* | 605511 |
| *TMTC2* | 615856 |
| *TNC* | 187380 |
| *TPRN* | 613354 |
| *TRIOBP* | 609761 |
| *TSPEAR* | 612920 |
| *USH1C* | 605242 |
| *USH1G* | 607696 |
| *USH2A* | 608400 |
| *WBP2* | 606962 |
| *WFS1* | 606201 |

**Additional File 1.** List of Exome panel evaluated genes with OMIM number.

| **Gene** | **OMIM number** |
| --- | --- |
| *ABHD12* | 613599 |
| *ACTG1* | 102560 |
| *ADCY1* | 103072 |
| *ADGRV1* | 602851 |
| *AIFM1* | 300169 |
| *ATP2B2* | 108733 |
| *BDP1* | 607012 |
| *BSND* | 606412 |
| *CABP2* | 607314 |
| *CCDC50* | 611051 |
| *CD164* | 603356 |
| *CDC14A* | 603504 |
| *CDH23* | 605516 |
| *CEACAM16* | 614591 |
| *CIB2* | 605564 |
| *CLDN14* | 605608 |
| *CLIC5* | 607293 |
| *CLRN1* | 606397 |
| *COCH* | 603196 |
| *COL11A2* | 120290 |
| *COL4A6* | 303631 |
| *CRYM* | 123740 |
| *DCDC2* | 605755 |
| *DFNA5* | 608798 |
| *DFNB31* | 607928 |
| *DFNB59* | 610219 |
| *DIABLO* | 605219 |
| *DIAPH1* | 602121 |
| *DIAPH3* | 614567 |
| *DMXL2* | 612186 |
| *EDN3* | 131242 |
| *EDNRB* | 131244 |
| *ELMOD3* | 615427 |
| *EPS8* | 600206 |
| *EPS8L2* | 614988 |
| *ESPN* | 606351 |
| *ESRRB* | 602167 |
| *EYA4* | 603550 |
| *FAM65B* | 611410 |
| *GIPC3* | 608792 |
| *GJB2* | 121011 |
| *GJB3* | 603324 |
| *GJB6* | 604418 |
| *GPSM2* | 609245 |
| *GRHL2* | 608576 |
| *GRXCR1* | 613283 |
| *GRXCR2* | 615762 |
| *HARS* | 142810 |
| *HGF* | 142409 |
| *HOMER2* | 604799 |
| *ILDR1* | 609739 |
| *KARS* | 601421 |
| *KCNQ4* | 603537 |
| *KITLG* | 184745 |
| *LHFPL5* | 609427 |
| *LOXHD1* | 613072 |
| *LRTOMT* | 612414 |
| *MARVELD2* | 610572 |
